# Plasma cardiac troponin T as a marker of skeletal muscle involvement in amyotrophic lateral sclerosis

**DOI:** 10.1093/braincomms/fcag358

**Published:** 2026-09-23

**Authors:** Linn Öijerstedt, Jianing Lin, Aditya Singh, Solmaz Yazdani, Annamari M Saarinen, Ulf Kläppe, Alexander Juto, Sanharib Chamoun, Sofia Imrell, Fang Fang, Sebastian A Lewandowski, Caroline Ingre

**Affiliations:** Department of Clinical Neuroscience, Karolinska Institutet, SE-171 77 Stockholm, Sweden; Department of Neurology, Karolinska University Hospital, SE-171 76 Stockholm, Sweden; Department of Clinical Neuroscience, Karolinska Institutet, SE-171 77 Stockholm, Sweden; Department of Neurology, Karolinska University Hospital, SE-171 76 Stockholm, Sweden; Center for Molecular Medicine, Karolinska Institutet, SE-171 77 Stockholm, Sweden; Department of Clinical Neuroscience, Karolinska Institutet, SE-171 77 Stockholm, Sweden; Department of Neurology, Karolinska University Hospital, SE-171 76 Stockholm, Sweden; Center for Molecular Medicine, Karolinska Institutet, SE-171 77 Stockholm, Sweden; Department of Clinical Neuroscience, Karolinska Institutet, SE-171 77 Stockholm, Sweden; Department of Clinical Neuroscience, Karolinska Institutet, SE-171 77 Stockholm, Sweden; Department of Neurology, Karolinska University Hospital, SE-171 76 Stockholm, Sweden; Center for Molecular Medicine, Karolinska Institutet, SE-171 77 Stockholm, Sweden; Department of Clinical Neuroscience, Karolinska Institutet, SE-171 77 Stockholm, Sweden; Department of Neurology, Karolinska University Hospital, SE-171 76 Stockholm, Sweden; Department of Clinical Neuroscience, Karolinska Institutet, SE-171 77 Stockholm, Sweden; Department of Neurology, Karolinska University Hospital, SE-171 76 Stockholm, Sweden; Department of Clinical Neuroscience, Karolinska Institutet, SE-171 77 Stockholm, Sweden; Department of Neurology, Karolinska University Hospital, SE-171 76 Stockholm, Sweden; Department of Neurology, Örebro University, SE-701 82 Örebro, Sweden; Institute of Environmental Medicine, Karolinska Institutet, SE-171 77 Stockholm, Sweden; Department of Clinical Neuroscience, Karolinska Institutet, SE-171 77 Stockholm, Sweden; Department of Neurology, Karolinska University Hospital, SE-171 76 Stockholm, Sweden; Center for Molecular Medicine, Karolinska Institutet, SE-171 77 Stockholm, Sweden; Department of Clinical Neuroscience, Karolinska Institutet, SE-171 77 Stockholm, Sweden; Department of Neurology, Karolinska University Hospital, SE-171 76 Stockholm, Sweden

**Keywords:** motor neuron disease, denervation, muscle, longitudinal, isoform

## Abstract

Plasma cardiac troponin T (cTnT) is widely used as a marker of myocardial injury, yet recent studies suggest elevations in amyotrophic lateral sclerosis (ALS). Whether these elevations reflect disease-associated skeletal muscle remodelling remains unresolved. In this translational study, we analysed longitudinal plasma cTnT in a large clinical cohort comprising 496 patients with ALS and 84 with benign fasciculation syndrome (BFS). To investigate the biological origin of circulating cTnT, we examined publicly available skeletal muscle transcriptomes from patients with ALS (*n* = 28) and healthy controls (*n* = 17), and quantified troponin isoform expression in skeletal muscle from *SOD1^G93A^* mice and wild-type littermates. Plasma cTnT was significantly higher in ALS than in BFS (median 18 versus 6 ng/L, *P* = 3.6 × 10^−27^), with 57% of patients with ALS exceeding the cardiologic threshold of 15 ng/L. Diagnostic performance was high [area under the curve (AUC) 0.87], with excellent specificity (94%). Longitudinal modelling revealed a progressive increase in cTnT in ALS whereas levels remained stable in BFS. Skeletal muscle transcriptomics demonstrated robust upregulation of *TNNT2*, encoding cardiac troponin T, in ALS muscle, while other troponin isoforms were unchanged. In *SOD1^G93A^* mice, severely affected gastrocnemius muscle exhibited isoform-specific remodelling, including 20-fold upregulation of *Tnnt2* and slow-fibre troponins, alongside downregulation of fast skeletal isoforms. Across clinical, transcriptomic, and experimental data, elevated plasma cTnT in ALS is best explained by disease-associated skeletal muscle remodelling rather than cardiomyocyte injury. Plasma cTnT emerges as a dynamic and accessible biomarker in ALS capturing muscle involvement. Prospective incorporation of cTnT into clinical trials and evaluation across broader neuromuscular differentials are warranted.

## Teaser

Öijerstedt *et al.* show elevated plasma cardiac troponin T and a progressive increase over time in patients with amyotrophic lateral sclerosis compared with benign fasciculation syndrome. Integrated clinical, transcriptomic, and experimental data indicate a skeletal muscle origin, supporting plasma troponin T as a biomarker of muscle involvement rather than myocardial injury.

## Graphical Abstract

**Graphical Abstract:**
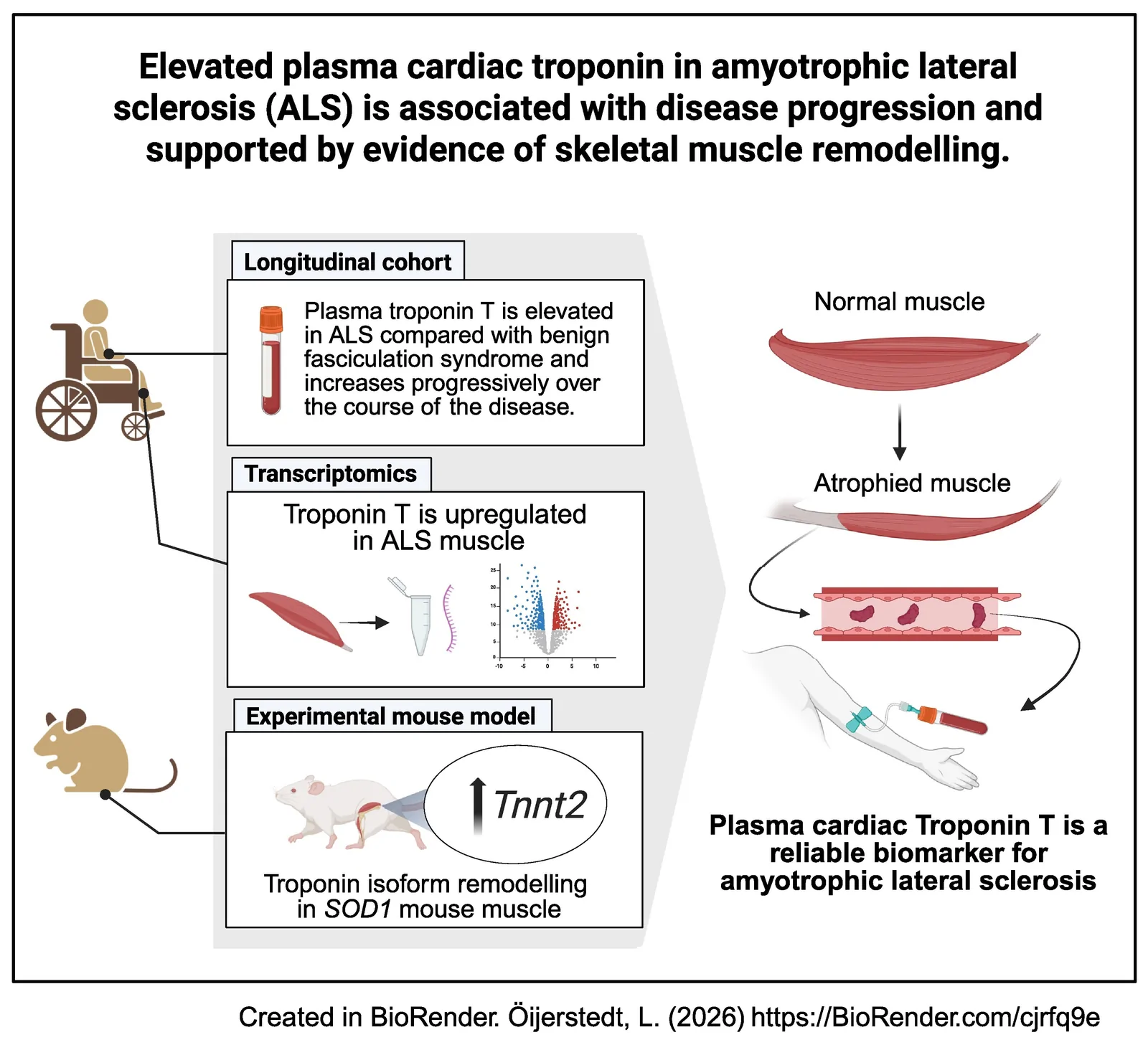

## Introduction

Amyotrophic lateral sclerosis (ALS) is a devastating disease without reliable biomarkers to support diagnosis and treatment monitoring. The disease is clinically heterogenic which contributes to prolonged diagnostic procedures, with a global diagnostic delay of ∼11 months.^1^ While neurofilament light chain (NfL) is currently the most widely used biomarker, reflecting axonal degeneration, it has limitations in capturing peripheral aspects of disease pathology.^2,3^ This has prompted interest in biomarkers that may better reflect muscle involvement and disease progression. One such candidate is cardiac troponin T (cTnT). Routinely used in clinical practice as a biomarker for the detection of myocardial injury, plasma cTnT has been found to be elevated in patients with ALS, even at early disease stages or near the time of diagnosis, sometimes before overt muscle weakness is clinically evident.^4-6^ Notably, these elevations often occur in absence of cardiac disease.

Previous studies in other neuromuscular disorders suggest that regenerating or diseased skeletal muscle can aberrantly express cardiac isoforms of troponin, including cTnT.^7,8^ This raises the possibility that elevated plasma cTnT in ALS reflects skeletal muscle remodelling rather than cardiomyocyte damage. Supporting this hypothesis, our recent work demonstrated that high plasma cTnT was correlated with lower motor neuron denervation, further implicating it as a marker of muscle involvement in ALS pathology.^9^

To explore the origin and clinical relevance of cTnT in ALS, we therefore complemented the project using a translational approach. We analysed longitudinal plasma cTnT levels in a large ALS cohort, including comparisons with patients diagnosed with benign fasciculation syndrome (BFS). In parallel, we examined publicly available skeletal muscle transcriptomes from patients with ALS and healthy controls and investigated troponin isoform expression in the *SOD1^G93A^* ALS mouse model. Our aims were to determine whether elevated plasma cTnT arises from disease-associated muscle remodelling and to evaluate its potential as a diagnostic and longitudinal biomarker in ALS.

## Materials and methods

### Clinical cohort

We retrospectively identified 497 patients with ALS and 85 with BFS from the Karolinska University Hospital database (December 2010–September 2024). Patients were classified based on ICD-10 coding (G12.2 for ALS; R25.3 for BFS). To ensure diagnostic validity, follow-up records were re-examined prior to analysis, with last verification performed on April 19, 2025. One individual who subsequently developed motor neuron disease (progressive muscular atrophy) during follow-up was excluded from all analyses. Details on patient selection and exclusion criteria are provided in the Supplementary Methods, Supplementary Fig. 1. Venous blood samples were collected during the clinical workup and analysed for cTnT. The laboratory analyses were performed at Karolinska University Laboratory using a high-sensitive assay (Roche Diagnostics Elecsys 5th generation, Rotkreuz, Switzerland). Samples obtained within 90 days of diagnosis were considered baseline. In total, 580 baseline and 1529 follow-up samples were included. The study was conducted in accordance with the Declaration of Helsinki. The study was reviewed and approved by the Swedish Ethical Review Authority (diary numbers 2017/1895-31 and 2018/1605-31).

### Human skeletal muscle transcriptomics

Transcriptomic data from ALS muscle biopsies (*n* = 28) and controls (*n* = 17) were obtained from an open-access cohort (Freydenzon *et al*., medRxiv 2023) https://doi.org/10.1101/2023.03.13.23287229.

Biopsies were collected from the vastus lateralis and processed with STAR and Salmon pipelines. Differential expression was analysed using DESeq2, correcting for sex. Further details are provided in the Supplementary Methods.

### Troponin isoform expression in mice

Transgenic *SOD1^G93A^* mice (*n* = 4) and wild-type (WT) littermates (*n* = 4) were sacrificed at around 140 days of age. At the time of sacrifice, *SOD1^G93A^* mice displayed advanced motor symptoms and marked gastrocnemius (GA) atrophy. GA and biceps muscles were dissected, snap frozen, and stored at −80 °C. RNA expression of troponin isoforms was quantified by qPCR, normalized to housekeeping transcripts. The levels of cTnT and cTnI were also measured in serum and muscle homogenate samples and normalized to their total protein. For additional information see Supplementary material, Supplementary Table 1.

### Statistical analysis

All data pre-processing, statistical analysis and illustrations were performed in R Studio version 4.3.1. Assumptions were assessed visually by residual plots (independence and equal variance) and normal probability plots (normality).

### Clinical cohort

Plasma cTnT was not normally distributed, and the variable was log2 transformed prior to statistical analysis. One outlier was identified and excluded (log2 cTnT > 4 standard deviations above median log2 cTnT). The difference in median plasma cTnT between patients with ALS and BFS was assessed by Wilcoxon rank sum test. The difference in mean age between the diagnostic groups was assessed by a two-sample *t*-test. Pearson’s chi-squared tests were used to investigate the differences in the distribution of sex and of individuals with cTnT values equal or above the threshold of 15 ng/L (no violation of expected count in each cell). To evaluate the diagnostic performance of plasma cTnT for distinguishing ALS from BFS, we constructed receiver operating characteristic (ROC) curves and calculated the area under the curve (AUC) with 95% confidence intervals (CI). Sensitivity, specificity, positive predictive value (PPV), and negative predictive value (NPV) were estimated at prespecified thresholds, including the cut-off for myocardial damage of 15 ng/L. In addition, the optimal threshold for discrimination between ALS and BFS was derived using the Youden index, and corresponding performance metrics were reported.

Logistic regression was applied to evaluate the association between cTnT and diagnosis including adjustments for age and sex. Diagnostic performance was evaluated by receiver operating characteristics (ROC) curve analysis, and optimal cut-offs were derived using the Youden index.

Follow-up was limited to 5 years after diagnosis to avoid sparse data at very long follow-up times. Individuals who continued to contribute longitudinal plasma samples beyond this time point were censored at 5 years, whereas individuals whose observation ended earlier were treated as having an event corresponding to end of follow-up. To account for potential informative censoring arising from differential dropout between ALS and BFS, longitudinal plasma cTnT trajectories were jointly modelled with time-to-end-of-follow-up outcomes. First, the time-to-event was modelled using a Cox proportional hazards model including diagnosis, age, and sex as covariates. In parallel, longitudinal plasma cTnT values were modelled using a linear mixed-effects model with fixed effects for age, sex, time from baseline, diagnosis, and interaction term between diagnosis and time from baseline. Subject-specific random intercepts and random slopes for time were included to account for within-individual correlation. The two sub models were then combined in a joint modelling framework, allowing the longitudinal cTnT process to inform the hazard of dropout and thereby reducing bias due to informative loss to follow-up. Model parameters were estimated using a Bayesian joint model fitted by Markov chain Monte Carlo sampling (12 000 iterations, burn-in 6000, thinning interval of four).

### Mice cohort

To assess differences in cardiac troponin levels between muscle type (GA versus biceps) and disease status (*SOD1* versus WT), a two-way ANOVA was applied, with the WT biceps group set as the reference condition. Comparisons were made between WT-GA, *SOD1*-biceps, and *SOD1*-GA relative to this reference. To correct for multiple testing, post hoc pairwise comparisons were adjusted using Tukey correction.

Differences in serum and muscle troponin levels between *SOD1^G93A^* and wild-type mice were investigated using two-sided Mann-Whitney U tests. All *P*-values were adjusted using the Benjamini-Hochberg method. Adjusted *P*-values < 0.05 were considered statistically significant.

## Results

### Elevated plasma cTnT in ALS

Plasma cTnT concentrations were higher in patients with ALS than in patients with BFS at diagnosis (median cTnT in ALS 18 ng/L compared with 6 ng/L in BFS, *P* = 3.6e−27). Elevated plasma cTnT values (≥15 ng/L) were found in 57.1% of ALS patients compared with only 5.9% of BFS patients (*P* = 1.3e−17) (Table 1, Fig. 1).

**Figure 1:**
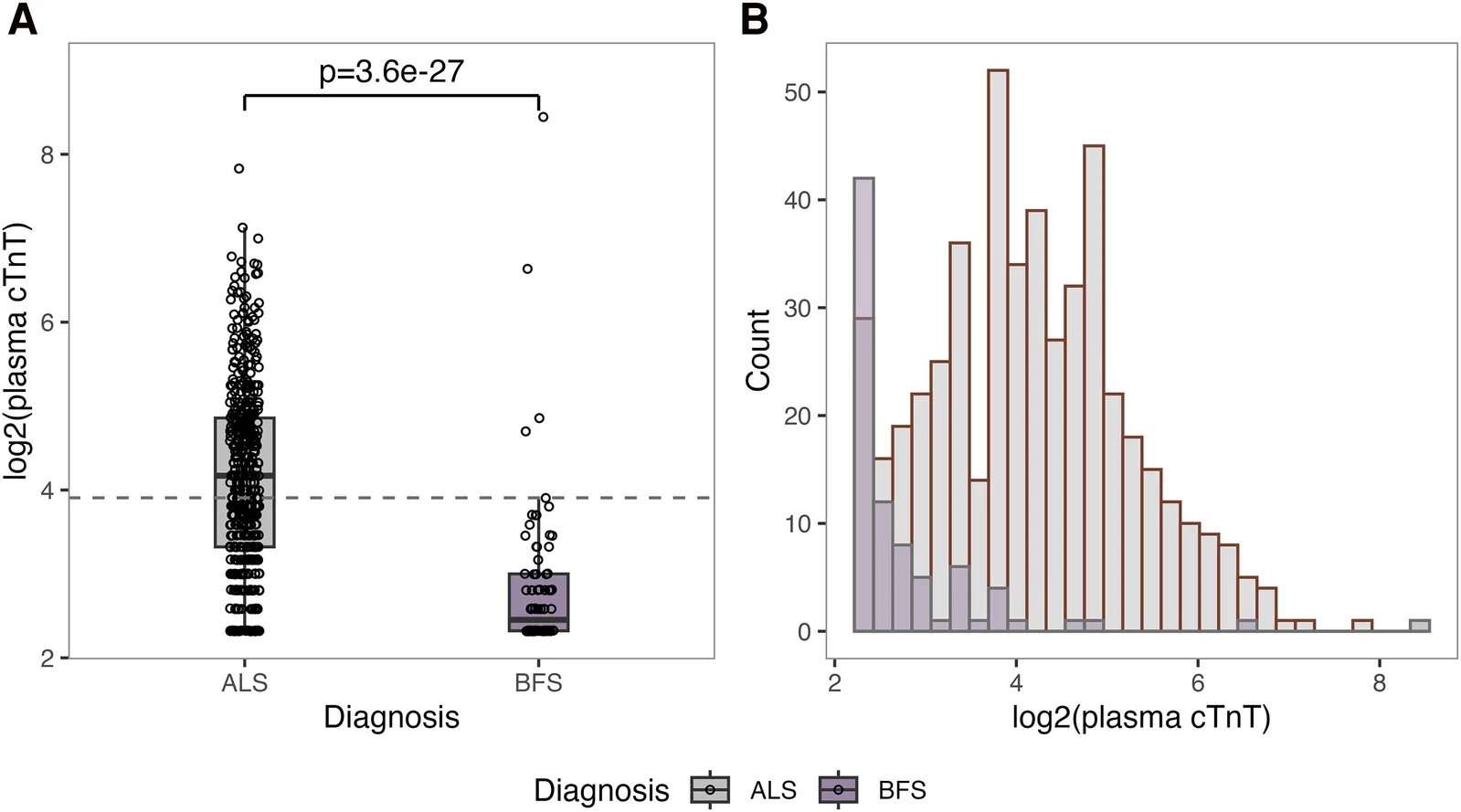
**Baseline plasma cardiac troponin T concentrations in patients with ALS and BFS.** (**A**) Boxplot of plasma cTnT concentrations in ALS (*N* = 496) and BFS (*N* = 84). The dashed line marks the cardiologic threshold of 15 ng/L (log2 = 3.9). Each data point represents one unique individual. Statistical test: Wilcoxon rank sum test. (**B**) Overlaid histograms of plasma cTnT concentrations show a rightward shift in the ALS distribution, indicating higher circulating troponin levels compared with BFS.

**Table 1:** Cohort characteristics of patients with ALS and BFS

	ALS (*N* = 496)	BFS (*N* = 84)	*P*-value
Age, mean years (SD)	67.0 (12.1)	51.8 (16.7)	2.6e−12^a^
Females, *N* (%)	216 (43.5)	38 (45.2)	0.87^b^
Plasma cTnT, median ng/L (IQR)	18 (19)	6 (3)	3.6e−27^c^
Plasma cTnT, *N* (%)			
No of individuals with ≥8.5 ng/L	410 (82.7)	17 (20.2)	2.2e−16^b^
≥ 15 ng/L	283 (57.1)	5 (5.9)	1.3e−17^b^

Baseline demographic variables and the proportion of individuals with elevated plasma cTnT (≥15 ng/L and ≥8.5 ng/L) within 90 days of diagnosis. ALS, amyotrophic lateral sclerosis; BFS, benign fasciculation syndrome; cTnT, cardiac troponin T; IQR, inter quartile range; L, litre; *N*, number; ng, nanogram; SD, standard deviation.

^a^Two sample *t*-test.

^b^Pearson’s chi-squared test.

^c^Wilcoxon rank sum test.

The diagnostic performance of plasma cTnT for distinguishing ALS from BFS was overall good, with an AUC of 0.87 (95% CI: 0.82–0.91). At the cardiologic threshold of 15 ng/L, the test demonstrated excellent specificity for ALS versus BFS (0.94, 95% CI: 0.88–0.99), such that fewer than 6% of patients with BFS would be incorrectly classified as ALS (Fig. 2). However, sensitivity at this threshold was modest (0.57, 95% CI 0.53–0.61), meaning that a substantial proportion of patients with ALS had cTnT values below 15 ng/L and would not be identified. Lowering the threshold to 8.5 ng/L (optimal cut-off determined by the Youden index) improved sensitivity to 0.83 (95% CI 0.79–0.86), albeit at the expense of reduced specificity (0.79, 95% CI 0.71–0.87). Importantly, the PPV remained very high at both thresholds (0.96 at 8.5 ng/L and 0.99 at 15 ng/L), underscoring the robustness of cTnT elevations in ALS when compared with BFS (Supplementary Table 2).

**Figure 2:**
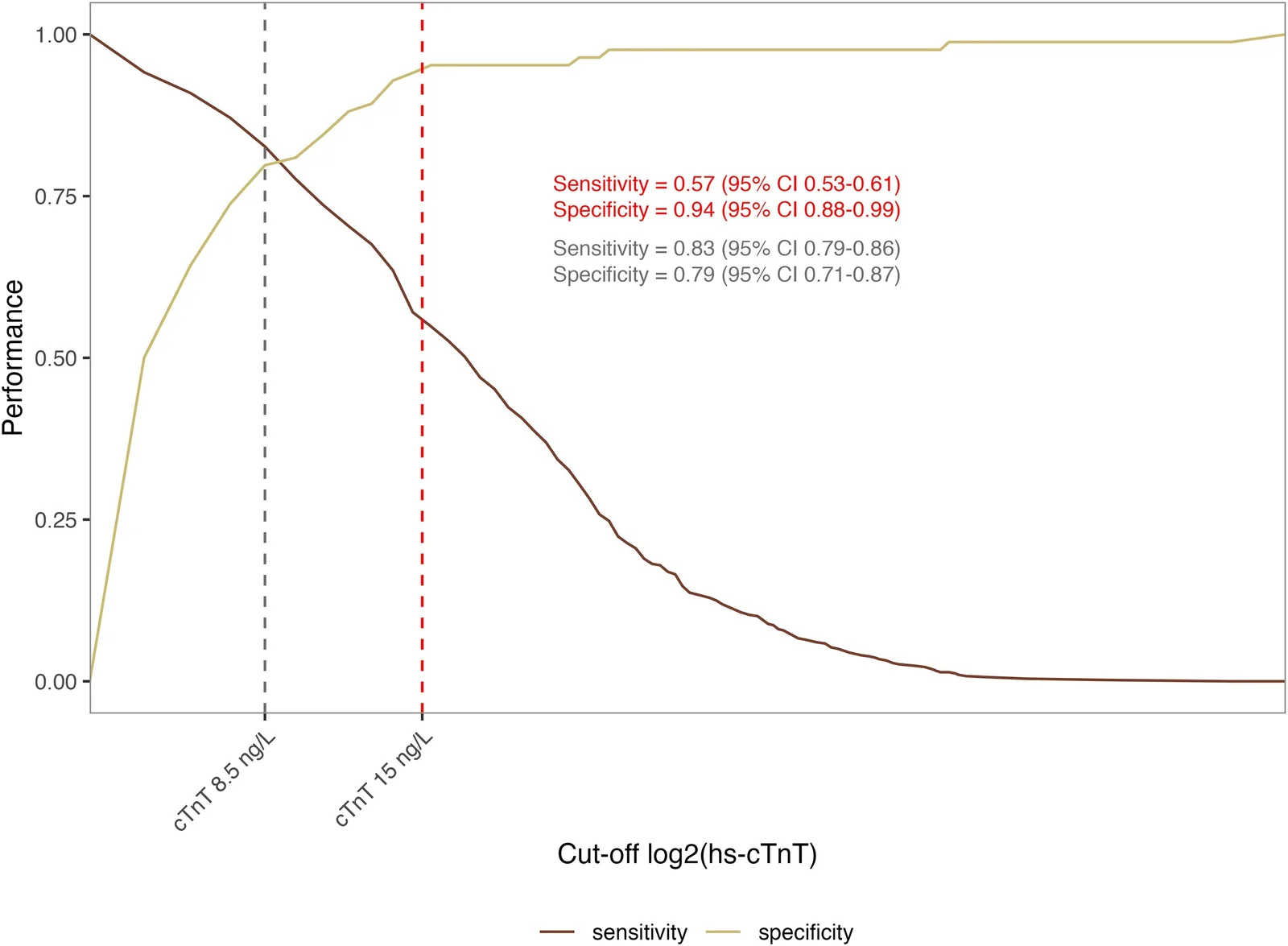
**Diagnostic performance of plasma cTnT in ALS versus BFS.** Sensitivity (brown line) and specificity (gold line) plotted as functions of plasma cTnT cut-off values (log2 scale). The vertical red dashed line corresponds to the cardiologic threshold of 15 ng/L (log2 = 3.95) and the grey dashed line to the cohort-derived cut-off (8.5 ng/L, log2 = 3.08) determined by the Youden index. Statistical test: ROC curve analysis. Sample size: ALS, *N* = 496 and BFS, *N* = 84. CI, confidence interval.

Logistic regression confirmed a strong and independent association between plasma cTnT higher than 15 ng/L and ALS (odds ratio 16.7, 95% CI 7.0–49.3), also after adjusting for age and sex.

A total of 2030 plasma samples were available for longitudinal analysis (580 patients, follow-up ≤5 years). Based on the joint model, plasma cTnT increased by an estimated 56.4% per year (95% CI: 34–78) in ALS, corresponding to an absolute rise of ∼10 ng/L per year (Fig. 3, Supplementary Table 3). In contrast, plasma cTnT remained essentially stable in BFS (−0.05 ng/L annual change corresponding to around −0.78% per year).

**Figure 3:**
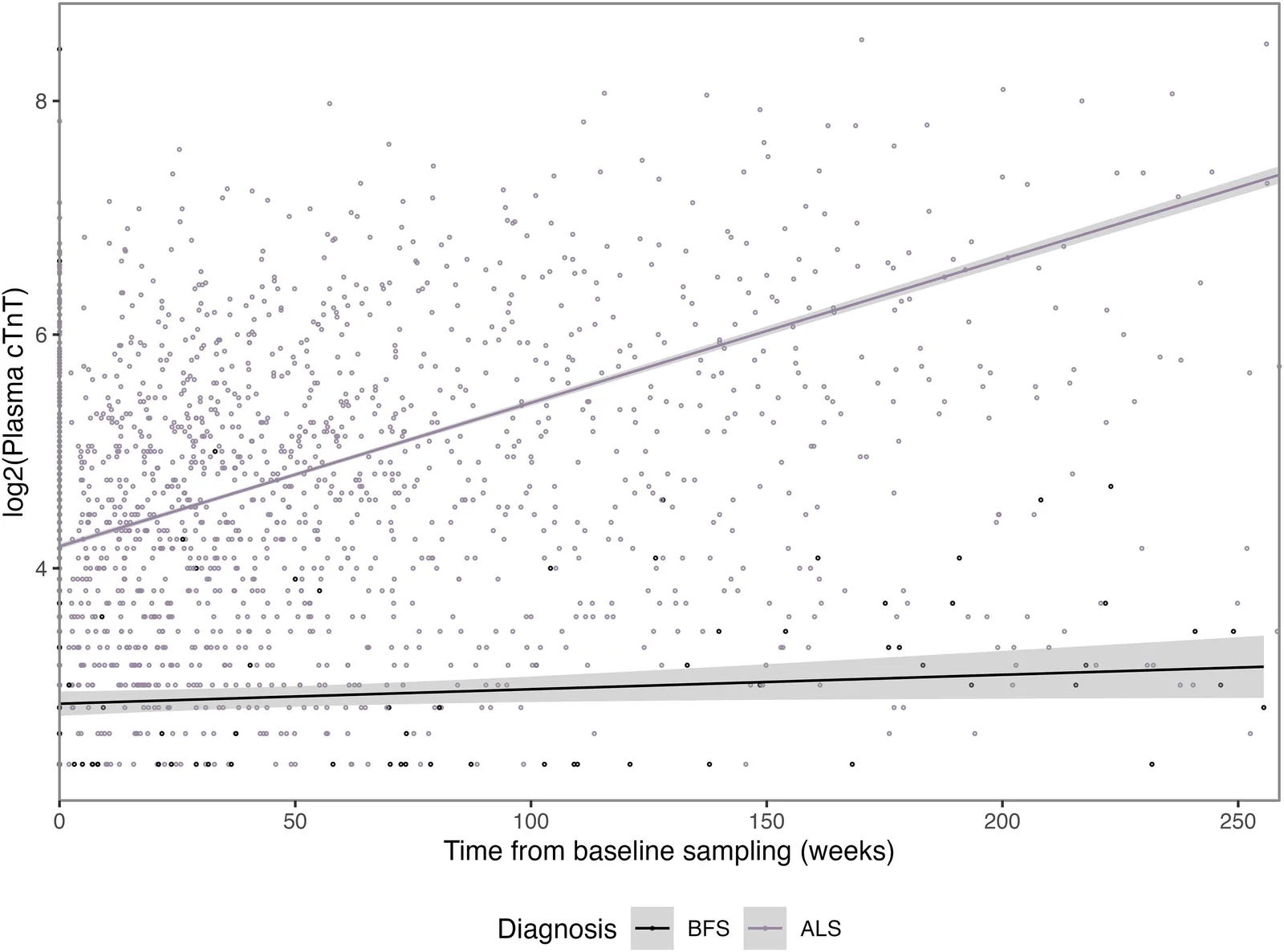
**Longitudinal trajectories of plasma cTnT in ALS and BFS.** Plasma cTnT trajectories over 5 years of follow-up derived from a joint model of longitudinal and survival data. Fixed effects were estimated using linear mixed-effects submodels, and statistical significance was assessed using Wald tests. Solid lines show model fit from model (grey = BFS, purple = ALS). Each point represents a cTnT measurement (ALS = 1885 observations, BFS = 145 observations). Shaded areas indicate 95% CI. Full model estimates and corresponding *P*-values are presented in Supplementary Table 3.

Diagnosis was not associated with differential dropout. However, higher contemporaneous plasma cTnT levels were associated with an increased hazard of end of follow-up, indicating that longitudinal cTnT trajectories were informative for dropout, supporting the use of a joint model.

### Up-regulation of *TNNT2* in human ALS muscle

Analysis of skeletal muscle biopsies from ALS patients and healthy controls confirmed disease-associated reprogramming of troponin isoform expression. Using both STAR- and Salmon-based quantifications, *TNNT2*, which encodes cardiac troponin T, was markedly up-regulated in ALS muscle relative to control muscle (Fig. 4, Supplementary Fig. 2). This signal was highly consistent across the two analytical pipelines, with fold-change estimates of 2.4-fold (Salmon) and 2.62-fold (STAR). By contrast, expression of other troponin isoforms (Table 2), both another cardiac troponin (*TNNI3*), the fast skeletal troponins (*TNNT3*, *TNNI2*) and slow skeletal isoforms (*TNNT1*, *TNNI1*), did not differ significantly between ALS and control samples.

**Figure 4:**
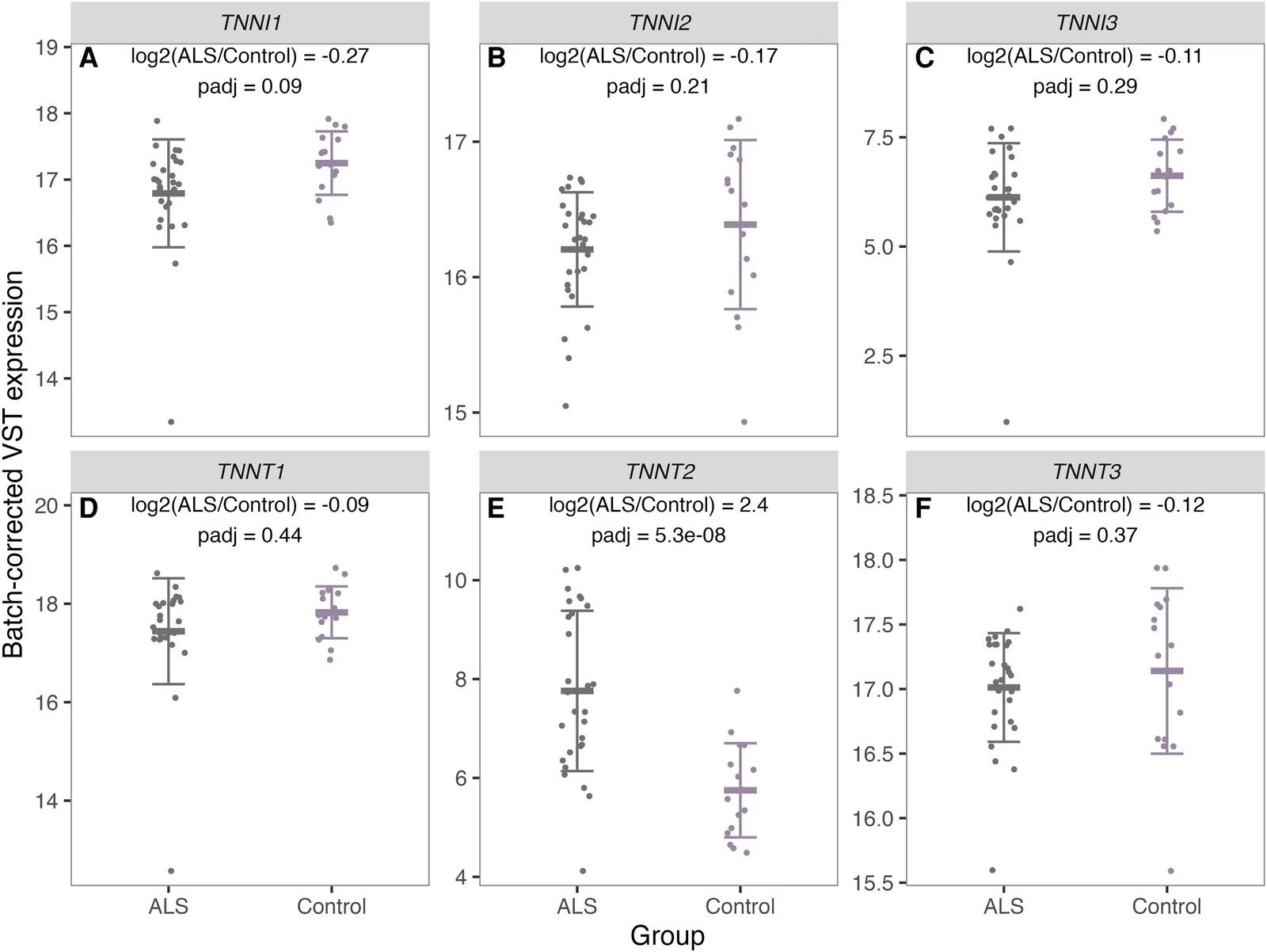
**Expression of troponin isoforms in human ALS versus control skeletal muscle.** Expression levels of troponin I (**A–C**) and troponin T (**D–F**) isoform genes. Each panel displays individual values, group means ± error bars, and the corresponding log_2_(ALS/Control) fold change with adjusted *P*-value (*P*adj) from differential expression testing (using DESeq2, based on negative binomial generalized linear models with Wald tests, including sex as a covariate). Sample size: ALS, *N* = 28 and Controls, *N* = 17. *P*adj, FDR adjusted *P*-value; VST, variance-stabilizing transformation.

**Table 2:** Troponin isoforms, associated genes, tissue expression and clinical biomarker relevance

Isoform	Gene (Human/Mouse)	Normal Expression	Clinical Biomarker Relevance
**Troponin T:** Anchors tropomyosin to thin filament			
Cardiac Troponin T	TNNT2/Tnnt2	Cardiac muscle	Widely used in clinical practice as a marker of myocardial injury
Fast Skeletal Troponin T	TNNT3/Tnnt3	Fast-twitch skeletal fibres	Not used clinically
Slow Skeletal Troponin T	TNNT1/Tnnt1	Slow-twitch skeletal fibres	Not used clinically
**Troponin I:** Inhibits actomyosin ATPase			
Cardiac Troponin I	TNNI3/Tnni3	Cardiac muscle	Used in cardiac diagnostics alongside cTnT
Fast Skeletal Troponin I	TNNI2/Tnni2	Fast-twitch skeletal fibres	Not used clinically
Slow Skeletal Troponin I	TNNI1/Tnni1	Slow-twitch skeletal fibres	Not used clinically

### Altered troponin isoform expression in ALS mice

Analysis of troponin isoform expression in *SOD1^G93A^* mice revealed muscle-specific remodelling patterns at end-stage disease. In the GA, which showed pronounced atrophy at sacrifice, there was a strong upregulation of *Tnnt2* compared with wild-type mice, with fold-changes exceeding 20 in three of the four animals (Fig. 5A). Cardiac Troponin I (*Tnni3*) was also increased in GA, but not in the biceps (Fig. 5B). A similar pattern was observed for isoforms associated with slow-twitch skeletal fibres (*Tnnt1*, *Tnni1*) which were increased in the GA but not in the biceps *SOD1^G93A^* mice (Fig. 5C and D). In contrast, fast skeletal troponin isoforms (*Tnnt3*, *Tnni2*) were reduced in *SOD1^G93A^* mice, in some cases to less than half of wild-type levels (Fig. 5E and F).

**Figure 5:**
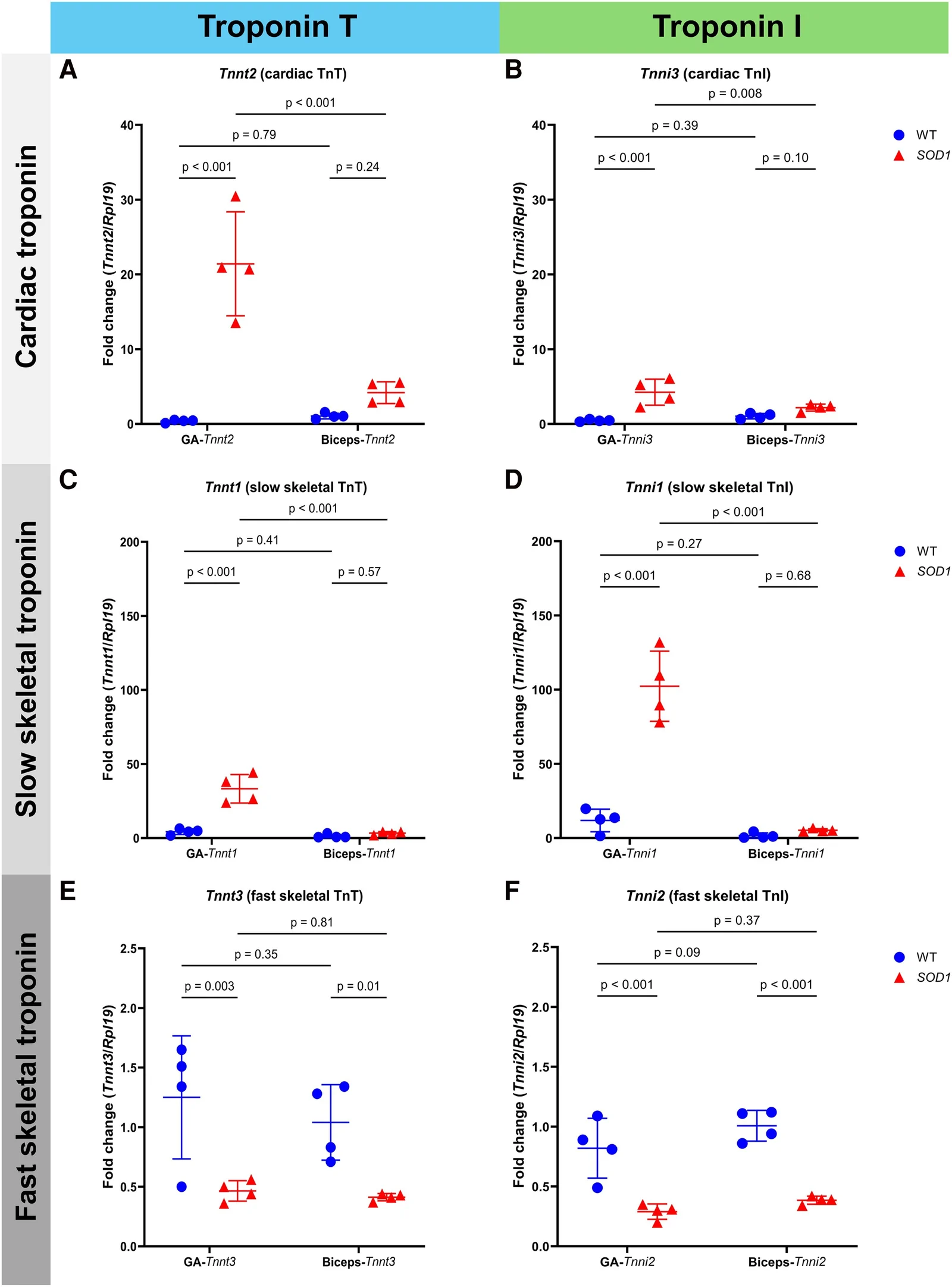
**Expression of troponin isoforms in skeletal muscle of *SOD1^G93A^* mice and wild-type controls.** Relative mRNA expression levels of cardiac (**A, B**), slow skeletal (**C, D**), and fast skeletal (**E, F**) troponin T and I isoforms in gastrocnemius and biceps muscles from wild-type (blue circles) and *SOD1^G93A^* (red triangles) mice (*n* = 4 per group). Expression values were normalized to mouse mitochondrial ribosomal protein L19 and are presented as mean ± standard error of the mean. *P*-values from two-way ANOVA. GA, gastrocnemius; mRPL19, mouse mitochondrial ribosomal protein L19; WT, wild-type.

In the biceps, which was relatively preserved at the time of collection, isoform remodelling was less pronounced. Fast skeletal troponin isoforms were reduced to a similar extent as in GA, but neither cardiac nor slow isoforms were significantly upregulated.

To assess whether the transcriptional changes translated to protein levels, we measured cTnT and cTnI in both serum and muscle homogenates using ELISA. In serum, cTnT levels were low and not increased in *SOD1^G93A^* mice compared with wild-type mice, while cTnI levels were below the detection threshold in all samples. In muscle tissue, neither cTnT nor cTnI levels differed significantly between *SOD1^G93A^* and wild-type mice in either GA or biceps, although cTnI levels in biceps showed greater variability in *SOD1^G93A^* mice (Supplementary Table 4).

## Discussion

In this translational study we combined clinical, molecular, and experimental approaches to investigate the origin and significance of elevated plasma cTnT in ALS. We analysed longitudinal plasma cTnT trajectories alongside human skeletal muscle transcriptomes and troponin isoform expression in the *SOD1^G93A^* mouse model. To our knowledge, this is the first study to integrate these complementary clinical and molecular data to explore a skeletal muscle contribution to circulating cTnT in ALS.

We demonstrate that plasma cardiac troponin T is elevated in ALS and discriminates ALS from BFS with high diagnostic accuracy. These findings are consistent with the report by Castro-Gómez *et al*. 2021, who likewise determined elevated plasma cTnT in ALS.^10^ Our data provide independent confirmation in an additional cohort and further strengthen the evidence that cTnT elevation is a reproducible feature of ALS. As patients with BFS lack electrophysiological evidence of denervation and show no clinical muscle atrophy, it represents a state of preserved muscle integrity despite overlapping symptoms, making it a suitable control for identifying ALS-specific changes. The ability of cTnT to distinguish ALS from BFS with high specificity supports an association with ALS-related disease processes. Importantly, longitudinal analysis revealed that cTnT increases progressively during follow-up, consistent with a dynamic biomarker of disease progression and confirms prior observations.^10,11^ These results align with emerging interventional data showing that Tofersen treatment stabilizes cTnT trajectories, suggesting that plasma cTnT reflects not only disease burden but also therapeutic response.^12^

At tissue level, the transcriptomic analyses provide biological support for our hypothesis of a skeletal muscle contribution. ALS muscle biopsies showed selective re-expression of *TNNT2*, the gene encoding cardiac troponin T, while other troponin isoforms were unchanged. In contrast, the *SOD1^G93A^* mouse model revealed a broader pattern of troponin remodelling in severely affected gastrocnemius muscles, with upregulation of cardiac and slow-skeletal troponins and downregulation of fast-skeletal isoforms. Notably, fold-changes for cardiac troponin T exceeded 20 in most animals, highlighting the magnitude of this response. This supports the view that skeletal muscle remodelling contributes to elevated plasma cTnT in ALS. However, the transcriptional changes were not accompanied by significant increases in cTnT or cTnI protein in mouse muscle or serum. The mouse data therefore demonstrate transcriptional remodelling of troponin isoforms but do not provide evidence of increased cardiac troponin protein abundance or release. The discordance may reflect biological differences between transcript and protein regulation, technical limitations of protein assays, or the rapidly progressive nature of the *SOD1^G93A^* model. Accordingly, these findings should be regarded as exploratory biological context rather than direct evidence for the source of circulating cTnT in patients. More broadly, this discordance between RNA and protein levels has been described in *SOD1* models, where marked induction of myogenic regulatory factor transcripts is not accompanied by corresponding protein increases at late disease stages, suggesting altered post-transcriptional or post-translational regulation in diseased muscle.^13^ These considerations indicate that tissue-level expression and circulating biomarker levels may not directly correspond and underscore the need for cautious interpretation when translating findings from experimental models to human disease. Importantly, the transcriptomic analyses were used to explore tissue-level mechanisms and are therefore intended to provide biological context for the human plasma findings rather than enable direct clinical comparisons between cohorts.

Taken together, these findings suggest that plasma cTnT may provide a widely accessible marker associated with skeletal muscle involvement in ALS. This contrasts with NfL, which primarily reflects neuroaxonal injury, and raises the possibility that the two biomarkers provide complementary information on different aspects of ALS pathology. The chronic and progressive trajectory of cTnT in ALS differs from the typical temporal pattern observed in acute myocardial injury and is compatible with an ongoing disease-associated process even if longitudinal behaviour alone cannot determine tissue origin.^10^ Unfortunately, NfL measurements were not available in the present patient cohort. However, we have conducted a follow-up study directly comparing plasma cTnT and cerebrospinal fluid NfL levels in a subset of patients with ALS, including a more detailed phenotypic characterization of the clinical cohort and analyses of their associations with troponin levels.^14^

Despite the strong performance of plasma cTnT in separating ALS from BFS, optimal thresholds in ALS remain unresolved. At the cut-off for cardiac damage of 15 ng/L, specificity approached 95%, ensuring that very few patients with BFS were misclassified as ALS. However, sensitivity at the same threshold was modest, highlighting the need to establish ALS-specific reference ranges, balancing sensitivity and specificity according to the intended clinical or research use. Notably, our cohort-derived threshold of 8.5 ng/L closely approximates the cut-off of 8.3 ng/L recently proposed by Lindenborn *et al*. 2026,^15^ providing remarkably consistent results across independent cohorts. This suggests that the clinically relevant reference range in ALS is substantially lower than the cardiologic threshold used to identify acute cardiac injury.

Although our data support skeletal muscle as the source of circulating cTnT in ALS, the precise cellular mechanisms remain speculative. Re-expression of cardiac troponin may reflect regenerative attempts, fibre-type switching toward slower oxidative phenotypes, or terminal stress responses in vulnerable fibres. Whether cardiac troponin isoforms contribute functionally to altered contractile properties or represent a by-product of transcriptional stress remains unknown. Future work combining transcriptomic, proteomic, and histological approaches will be needed to clarify these processes.

While our data provide converging evidence consistent with a skeletal muscle contribution to circulating cTnT, cardiac comorbidity cannot be formally excluded on an individual basis, as data on cardiac symptoms were not available in our clinical cohort, although cTnT samples collected for suspicion of acute myocardial infarction were excluded. Furthermore, plasma cTnI measurements were not available. However, the transcriptomic data show that *TNNI3* was not significantly upregulated in ALS muscle. In addition, Castro-Gómez *et al*. 2021 reported that plasma cTnI is rarely elevated in ALS despite increased cTnT levels.^10^ Together, these observations argue against a generalized myocardial injury signal as the primary explanation for elevated cTnT in ALS. Several other limitations also need considerations. A key limitation of this study is the lack of animal models that fully recapitulate all aspects of ALS phenotypes observed in patients. The *SOD1^G93A^* mouse model relies on mutant *SOD1* overexpression, which distinguishes it from most human ALS cases. Despite this limitation, the *SOD1^G93A^* model provides a robust and reproducible system for studying mechanisms associated with motor neuron degeneration and muscle atrophy. Consequently, the severity and rapid kinetics of neurodegeneration observed in this fast-progressing model likely reflect exaggerated proteotoxic stress rather than the full spectrum of ALS pathophysiology.

In our clinical cohort, we lacked systematic data on ALS progression and other clinical characteristics. In addition, we cannot fully exclude non-cardiac tissue sources other than skeletal muscle, and further investigations will be required to clarify potential extra-muscular contributions which were beyond the scope of this study. Moreover, our analyses of human skeletal muscle were limited to transcriptomics and did not include direct protein validation. Ongoing immunohistochemistry and proteomic studies in our lab will address this gap. Finally, while BFS represents a relevant comparator, additional disease controls, such as inflammatory or hereditary myopathies, are needed to further establish the specificity of cTnT for ALS.

## Conclusion

Plasma cardiac troponin T is elevated in ALS and increases longitudinally during follow-up. Human skeletal muscle transcriptomic data, supported by findings in the *SOD1^G93A^* model, demonstrate disease-associated remodelling of troponin isoform expression and are consistent with skeletal muscle contributing to circulating cTnT. Future studies should define the specificity of plasma cTnT across neuromuscular disorders and optimize its use for monitoring disease progression and treatment response.

## Supplementary Material

fcag358_Supplementary_Data

## Data Availability

The clinical dataset used and analysed during the current study are available from the corresponding author upon reasonable request. Code used for bulk RNA-seq analysis and data visualization is available via https://github.com/JianingLin96/2026-ALS-troponin-project.
