# Supplementary material for: Plasma cardiac troponin T as a marker of skeletal muscle involvement in amyotrophic lateral sclerosis
Source: Brain Commun. 2026 Sep 23;8(5):fcag358. doi: 10.1093/braincomms/fcag358 (PMC13648872; doi:10.1093/braincomms/fcag358)
Supplement: Supplementary file 1 [file fcag358_supplementary_data.docx]

# SUPPLEMENTARY MATERIAL

## Plasma cardiac troponin T as a marker of skeletal muscle involvement in amyotrophic lateral sclerosis

## Method

### Clinical cohort

We included 497 patients with ALS and 85 individuals with benign fasciculation syndrome (BFS). This part was performed as a retrospective study using the Karolinska University Hospital electronic database. Data was extracted from electronic health records over the period of almost 14 years, from December 2010 to September 2024 (see flow chart below). Patients were identified by searching for the ICD-10 codes G12.2 (motor neuron disease/ALS) and R25.3 (fasciculations). Patients recorded as G12.2 alone or in combination with R25.3 were considered MND/ALS, those with only R25.3 and never G12.2 were considered benign fasciculations. We included patients with age above 18 years and available cardiac troponin T plasma levels (N=858). Plasma cardiac Troponin T (cTnT) within 90 days of the first record of the clinical diagnosis (i.e. G12.2 or R25.3) was considered baseline and all subsequent samplings were considered follow-ups. Individuals with no sampling within 90 days of the first diagnosis were excluded (N=193). Analysis of plasma cTnT in an acute setting related to suspicion of myocardial incidence were also excluded (N=83). All records were anonymised prior to analysis, and the available clinical information was date of birth, sex, date of sampling, diagnosis, and date of diagnosis.

**Supplementary Figure 1. Flow chart of cohort selection.**

### Skeletal muscle transcriptomics

Skeletal muscle transcriptomic data were obtained from an open-access cohort described by Freydenzon and colleagues^1^ (medRxiv. 2023; <https://doi.org/10.1101/2023.03.13.23287229>).

According to the description of the original study, skeletal muscle biopsies were collected from the musculus vastus lateralis of 28 ALS patients and 17 healthy controls. Participants were recruited at the Royal Brisbane and Women’s Hospital motor neuron disease (MND) clinic. Eligible patients were required to meet the revised El Escorial criteria for a diagnosis of ALS at the time of biopsy. Healthy controls were recruited from unaffected relatives, friends, or community volunteers. Individuals with a history of diabetes mellitus or metabolic disease were excluded to minimize confounding by systemic metabolic conditions. Biopsies were performed from musculus vastus lateralis in the least clinically affected leg, using a Bergström needle modified for suction. Approximately 200 mg of tissue was collected per participant. RNA was extracted and assessed for integrity, with an average RNA Integrity Number (RIN) of 8.2 across the dataset, consistent with high-quality input material. Gene read counts generated using STAR (v2.7.0) and transcript abundances generated using Salmon (v1.0) were obtained from the original study and processed in R. Differential gene expression analysis was performed using DESeq2, with sex included as a covariate. For visualization, variance-stabilizing transformation (VST) was applied to the gene expression data, followed by covariate correction using ComBat^2^. Covariate-corrected VST values of genes of interest were used to generate gene expression dot plots, which were annotated with differential expression statistics. A more detailed description of the method is described in the original paper.

### Mouse studies

All animal experiments were approved following the Swedish National Board on research animal ethics and Stockholm Animal Research Ethics Committee under the ethical permit Dnr 23561-2022. Transgenic SOD1 G93A mice (The Jackson Laboratory, #004435, n=4) and wild-type littermate controls (n=4) were used to assess muscle-specific troponin isoform expression at end-stage disease. The breeding colony was maintained under standard housing conditions with ad libitum access to food and water. Wild-type mice were sacrificed at the age of 138 days, with body weights ranging from 28.3 to 31.4 g. SOD1 G93A mice were sacrificed at a median age of 141 days (range 140–142) and had lower body weights at sacrifice (22.3 to 26.9 g). consistent with disease progression. At the time of sacrifice, all SOD1 G93A had a fulminant phenotype. Mice were anesthetized according to institutional guidelines, and blood was collected via intracardiac puncture using a 1 mL syringe fitted with a 23-gauge needle. Whole blood was transferred and allowed to clot for 30 minutes. Samples were then centrifuged at 2000 × g for 15 minutes at 4°C, and the serum supernatant was carefully collected and preserved at −80°C until further analysis. Gastrocnemius and biceps muscles were collected bilaterally immediately after sacrifice. Samples from one side were snap-frozen on dry ice in pre-cooled cryotubes for protein analysis, while contralateral muscles were preserved in RNAlater solution (Invitrogen. #AM7020) for subsequent RNA extraction. All samples were subsequently transferred to −80 °C for storage until processing.

### RNA expression analysis

Total RNA was extracted from frozen muscle samples using the Qiagen RNeasy mini kit (Qiagen, #74704), with genomic DNA removal with the RNase-free DNase set (Qiagen, #79254). Subsequently, cDNA was synthesized using a cDNA synthesis kit (BioRad Laboratories, #1708891), followed by qPCR reaction with SYBR green (BioRad Laboratories, #1708886) according to the manufacturer’s instructions. Gene expressions were calculated based on the 2−ΔΔCt method and was normalized to housekeeping Rpl19 gene transcripts (**Supplementary Table 1**).

cTnT and cTnI levels in muscle and serum samples were measured using the Mouse Cardiac Troponin T ELISA Kit (antibodies.com, A5377) and the Mouse Cardiac Troponin I ELISA Kit (antibodies.com, A1881), respectively. Muscle sample homogenates collection and ELISA assays was performed following protocol instructions. Briefly, serum or tissue lysate samples diluted in assay diluent were added to the pre-coated microtiter plate wells, along with serial dilutions of the provided standards. Liquid was aspirated after a 2-hour incubation, followed by subsequent detection solution incubations and multiple washing steps following protocol instructions. At last, the substrate solution was added for a recommended incubation period, and the reaction was stopped with stop solution. The optical density was measured at 450nm with a microplate reader. Four-parameter logistic curves were fitted to duplicate standard samples and used to calculate concentrations in the test samples. For diluted samples, concentrations obtained from the standard curve were multiplied by the corresponding dilution factor. Total protein concentration in serum and muscle homogenates was measured using a BCA Protein Assay Kit (Thermo Scientific, #23225). Levels of cTnT and cTnI were normalized to total protein and reported as pg per mg of total protein.

**Supplementary Table 1.** qPCR primers and primer blast.

| Targeted genes | Name | Primers | Product length | Targeted sequence |
| --- | --- | --- | --- | --- |
| **Slow skeletal TnI** | Tnni1-F | ATGCCGGAAGTTGAGAGGAAA | 140 | [NM_021467.5](https://www.ncbi.nlm.nih.gov/entrez/viewer.fcgi?db=nucleotide&id=162951863) |
|  | Tnni1-R | TCCGAGAGGTAACGCACCTT |  |  |
| **Fast skeletal TnI** | Tnni2-F | AGAGTGTGATGCTCCAGATAGC | 170 | [NM_009405.3](https://www.ncbi.nlm.nih.gov/entrez/viewer.fcgi?db=nucleotide&id=1395168509) |
|  | Tnni2-R | AGCCACGTCGATCTTCGCA |  |  |
| **Cardiac TnI** | Tnni3-F | AGGAGATGGAACGAGAGGCAGAAG | 129 | [NM_009406.4](https://www.ncbi.nlm.nih.gov/entrez/viewer.fcgi?db=nucleotide&id=1134612221) |
|  | Tnni3-R | CGTGAAGCTGTCGGCATAAGTCC |  |  |
| **Slow skeletal TnT** | Tnnt1-F | CCCCCGAAGATTCCAGAAGG | 154 | 5 variants |
|  | Tnnt1-R | TGCGGTCTTTTAGTGCAATGAG |  |  |
| **Cardiac TnT** | Tnnt2-F | GAGACAGAGGAGGCCAACGTA | 338 | 9 variants |
|  | Tnnt2-R | CTTTCCTTCTCCCGCTCAT |  |  |
| **Fast skeletal TnT** | Tnnt3-F | GGAACGCCAGAACAGATTGG | 104 | 17 variants |
|  | Tnnt3-R | TGGAGGACAGAGCCTTTTTCTT |  |  |
| **Housekeeping gene** | Rpl19-F | CCGGCTTCTCAGGAGATAC | 103 | [NM_009078.2](https://www.ncbi.nlm.nih.gov/entrez/viewer.fcgi?db=nucleotide&id=226958652)  [NM_001159483.1](https://www.ncbi.nlm.nih.gov/entrez/viewer.fcgi?db=nucleotide&id=226958656) |
|  | Rpl19-R | GATGCGCTTGTTTTTGAACAC |  |  |

## Results

**Supplementary Table 2. Diagnostic performance of plasma cTnT in ALS versus BFS.**

| Plasma cTnT threshold (ng/L) | Sensitivity | Specificity | PPV | NPV |
| --- | --- | --- | --- | --- |
| 8.5 | 0.83 | 0.79 | 0.96 | 0.44 |
| 15 | 0.57 | 0.94 | 0.99 | 0.27 |

**Supplementary Table 3. Estimates from the joint model.** SD, standard deviations; CI, confidence interval.

| **Variable** | **Estimate** | **SD** | **CI low** | **CI high** | **p** | **Rhat** | **Model** |
| --- | --- | --- | --- | --- | --- | --- | --- |
| **Fixed effects** | | | | | | | longitudinal |
| (Intercept) | 1.24E+00 | 2.21E-01 | 8.25E-01 | 1.68E+00 | <0.001 | 1.24E+00 |  |
| Age | 2.34E-02 | 3.51E-03 | 1.63E-02 | 3.00E-02 | <0.001 | 2.34E-02 |  |
| Male sex | 7.03E-01 | 8.23E-02 | 5.46E-01 | 8.68E-01 | <0.001 | 7.03E-01 |  |
| Months from baseline | -2.19E-04 | 1.84E-03 | -3.87E-03 | 3.42E-03 | 9.14E-01 | -2.19E-04 |  |
| ALS diagnosis | 9.91E-01 | 1.28E-01 | 7.44E-01 | 1.25E+00 | <0.001 | 9.91E-01 |  |
| Months from baseline:  ALS diagnosis | 1.26E-02 | 1.96E-03 | 8.82E-03 | 1.66E-02 | <0.001 | 1.26E-02 |  |
| ALS diagnosis | -1.59E+00 | 2.74E-01 | -2.11E+00 | -1.05E+00 | <0.001 | -1.59E+00 | survival |
| Age | 2.43E-04 | 4.71E-03 | -8.71E-03 | 9.62E-03 | 9.79E-01 | 2.43E-04 |  |
| Male sex | -7.28E-01 | 1.17E-01 | -9.59E-01 | -5.01E-01 | <0.001 | -7.28E-01 |  |
| Plasma hs-cTnT | 9.44E-01 | 5.86E-02 | 8.27E-01 | 1.06E+00 | <0.001 | 9.44E-01 |  |
| **Random effects** | Variance | SD |  |  |  |  |  |
| Intercept (id) | 0.91 | 0.95 |  |  |  |  |  |
| Slope (time:id) | 0.00008 | 0.009 |  |  |  |  |  |
| Residual (within-subject) | 0.09 | 0.30 |  |  |  |  |  |

**Supplementary Figure 2.** **Expression of troponin isoforms in human ALS versus control skeletal muscle (STAR).** Expression levels of troponin I (**A-C**) and troponin T (**D-F**) isoform genes. Each panel displays individual values, group means ± error bars, and the corresponding log₂(ALS/Control) fold change with adjusted p-value (padj) from differential expression testing (using DESeq2, based on negative binomial generalised linear models with Wald tests, including sex as a covariate). Sample size: ALS, N=28 and Controls, N=17.

**Supplementary Table 4. Results from protein analysis in serum and muscle in mice.** WT, wild type; n.d., not detected. Unit: pg/mg.

|  | WT | WT | WT | WT | SOD1 | SOD1 | SOD1 | SOD1 | p-value |
| --- | --- | --- | --- | --- | --- | --- | --- | --- | --- |
| Serum-cTnT | 0.64 | 1.21 | 0.80 | 0.49 | 0.46 | 0.66 | 0.19 | 0.71 | 0.34 |
| Serum-cTnI | n.d. | n.d. | n.d. | n.d. | n.d. | n.d. | n.d. | n.d. | NA |
| GA-cTnT | 146.82 | 331.46 | 343.50 | 302.57 | 168.89 | 126.39 | 166.41 | 165.73 | 0.25 |
| Biceps-cTnT | 75.37 | 63.37 | 92.55 | 68.22 | 377.48 | 167.83 | 202.81 | 133.73 | 0.071 |
| GA-cTnI | 295.28 | 393.28 | 369.15 | 344.69 | 173.15 | 272.82 | 336.74 | 311.04 | 0.19 |
| Biceps-cTnI | 179.13 | 164.99 | 183.08 | 161.39 | 3295.72 | 413.70 | 519.00 | 456.34 | 0.71 |

## References

1.	Freydenzon A, Wani S, Bharti V, et al. RNA-seq analysis of skeletal muscle in motor neurone disease cases and controls. *medRxiv*. Published online March 19, 2023:2023.03.13.23287229. doi:10.1101/2023.03.13.23287229

2.	Leek JT, Johnson WE, Parker HS, Jaffe AE, Storey JD. The sva package for removing batch effects and other unwanted variation in high-throughput experiments. *Bioinformatics*. 2012;28(6):882-883. doi:10.1093/BIOINFORMATICS/BTS034
